# A transcriptomics analysis of the Tbx5 paralogues in zebrafish

**DOI:** 10.1371/journal.pone.0208766

**Published:** 2018-12-10

**Authors:** Erin A. T. Boyle Anderson, Robert K. Ho

**Affiliations:** 1 Committee on Development, Regeneration, and Stem Cell Biology, University of Chicago, Chicago, Illinois, United States of America; 2 Department of Organismal Biology and Anatomy, University of Chicago, Chicago, Illinois, United States of America; Deakin School of Medicine, AUSTRALIA

## Abstract

TBX5 is essential for limb and heart development. Mutations in TBX5 are associated with Holt-Oram syndrome in humans. Due to the teleost specific genome duplication, zebrafish have two copies of TBX5: *tbx5a* and *tbx5b*. Both of these genes are expressed in regions of the lateral plate mesoderm and retina. In this study, we perform comparative RNA sequencing analysis on zebrafish embryos during the stages of lateral plate mesoderm migration. This work shows that knockdown of the Tbx5 paralogues results in altered gene expression in many tissues outside of the lateral plate mesoderm, especially in the somitic mesoderm and the intermediate mesoderm. Specifically, knockdown of *tbx5b* results in changes in somite size, in the differentiation of vasculature progenitors and in later patterning of trunk blood vessels.

## Introduction

The first Tbox gene identified was *T* (also known as *brachyury*), which was found to be a tissue specific transcription factor[1] essential for mesoderm formation. An entire family of genes has now been identified which contains the Tbox domain, a highly conserved region of 174–186 amino acids[2] that binds DNA in a sequence-specific manner[3,4].

*Tbx5* in amniotes is a Tbox gene that plays a role in development of both heart and limb. It is expressed in a territory of the lateral plate mesoderm (LPM), a thin tissue lying lateral to the somitic mesoderm, as well as in the eye. The more anterior *Tbx5*-positive cells in the LPM contribute to the secondary heart field and other tissues, while the more posterior *Tbx5*-positive LPM cells contribute to the forelimb. In humans, mutation of just one copy of *TBX5* is associated with Holt-Oram syndrome, in which heart and forearm defects occur in 1 in 100,000 live births[5]. These heart defects include septation defects and cardiac conduction syndrome. The limb defects have a range of severity, from minor defects in the thumb, to truncations of large parts of the arm, with a reported bias towards more prevalent defects on the anterior part of the limb[6].

Mice that are haploinsufficent for *Tbx5* mimic the Holt-Oram syndrome phenotype, having both defective heart and forelimb tissues[7]. Mice completely lacking *Tbx5* die early due to implantation defects caused by *Tbx5’*s role in extraembryonic tissues. However when Cre-mediated deletion is used to remove *Tbx5* from later-staged embryos, mice survive until embryonic day 10.5, although development stalls at day 9.5[7]. The heart tube of these mutant mice fails to undergo looping and forms only one atrium, rather than the normal two atria which should be present at this stage[7]. When *Tbx5* is removed selectively from tissue that will become the forelimbs allowing the mouse to continue development past embryonic day 9.5, those mice fail to form forelimb buds, leading to the complete lack of forelimbs in the perinatal mouse[8].

Due to the whole genome duplication in the lineage leading to teleost fish, zebrafish have two paralogous gene copies of *TBX5*: *tbx5a* and *tbx5b* [9]. There are high levels of conservation at the amino acid level in the Tbox domain between the two paralogues but there are low levels of conservation throughout the rest of the protein [10]. In the zebrafish, *tbx5a* is expressed in both the heart and limb field regions of the LPM, as well as the retina [11]. The sister paralogue *tbx5b* is expressed in both the retina and the heart field, a subset of where *tbx5a* is expressed [9]. While *tbx5a* is detectable through *in situ* hybridization as early as 14 hpf in the LPM, *tbx5b* is not detectable until 17 hpf [9]. Expression in the heart is similar for both paralogues until 36 hpf, when *tbx5b* expression is restricted to the ventricle, whereas *tbx5a* expression remains in both chambers of the heart [9]. Some studies report that there does not appear to be detectable expression of *tbx5b* distinct from background in the pectoral fin bud [9], while others have reported low levels of *tbx5b* expression in the resulting pectoral fins [10]. Despite this, Tbx5b-deficient embryos exhibit clear phenotypes in fin bud formation and fin development, indicating that it may function at some point in the developing fin field or surrounding tissues.

Tbx5a -/- mutants (*heartstring*) undergo normal heart jogging—the first break in right-left asymmetry—at 24 hpf [12]. However, heart looping does not occur until after 5 days post fertilization (dpf) [12]. Tbx5b-deficient embryos display earlier and more severe heart defects than *tbx5a* mutants. Tbx5b-deficient embryos display defects in heart development as early as 24 hours, as heart jogging fails to occur correctly, often resulting in a heart at the midline rather than on the left—although occasionally heart jogging occurs to the right [13]. Heart looping also fails to occur in Tbx5b-deficient embryos[13].

*Tbx5a* mutants (*heartstring*) do not develop a pectoral fin bud or pectoral fins. This is due to downstream loss of *fgf24* expression, which is regulated by Tbx5a and required for the migrating LPM cells to converge and form a fin bud[14,15]. Unlike Tbx5a-deficient embryos, Tbx5b-deficient embryos do form pectoral fin buds and fin, although they exhibit delayed fin bud formation and the resulting pectoral fins are often small or upturned, with misshapen edges[10]. Fin development appears to be delayed as the 48 hpf pectoral fins of Tbx5b morpholino-injected embryos appear morphologically similar to 36 hpf control embryos’ fins [13]. Pi-Roig et al hypothesize that *tbx5a* initiates fin outgrowth while *tbx5b* is required later in pectoral fin development to maintain this growth [13]. However, Tbx5b-deficient embryos still have delayed fin bud development which can be seen as early as 30 hpf, when the limb bud first becomes morphologically distinct in wildtype embryos [10]. This suggests that *tbx5b* may not exclusively function late in fin development, but may have earlier effects as well.

Although morpholino injections to either *tbx5* paralogue produce both heart and fin defects in zebrafish, mRNA can rescue the cognate phenotype, but not that of its paralogue [10], suggesting that there are functional differences between the two paralogues. Additionally, the Tbx5 paralogues differentially affect gene expression during development of both the heart and fins. Specifically, Tbx5a-deficient embryos lose expression of *fgf24* in the fin field while Tbx5b-deficient embryos maintain expression of *fgf24*, but show a delay in switching expression of *fgf24* from mesenchymal tissue to ectodermal tissue at 36 hpf[13]. In the heart, expression of *vcana* is expanded in embryos deficient in either Tbx5a or Tbx5b, but expression of *bmp4* is only restricted in embryos deficient in Tbx5a, not in embryos deficient in Tbx5b[10]. These changes, as well as changes in expression of other molecular markers, suggest that *tbx5a* and *tbx5b* may act within different transcriptional networks.

As previously stated, *tbx5a* and *tbx5b* result from a duplication event during the teleost specific genome duplication [10]. How have they evolved to have the possibly non-overlapping functions they have today, implied by the different effects of depletion and the fact that they cannot rescue their paralogue? As transcription factors, we can possibly gain insight into how the Tbx5 paralogues are functioning by understanding the transcriptional networks that they affect. In this paper, we perform comparative RNA sequencing experiments between the Tbx5 paralogues. In order to target genes that might play a role in the migration of limb bud and heart precursors, we collected embryos at 18 hpf and 21 hpf, during the time that the cells in the LPM are migrating. Our screen identified genes that are expressed (and differentially regulated) outside of the LPM, with two tissues of note being the vasculature and the somites. We also report the creation of a *tbx5b* mutant, which appears to validate the *tbx5b* morpholino phenotype.

## Methods

### Zebrafish embryos, morpholino injection and in situ hybridization

All zebrafish experiments were performed under the University of Chicago Institutional Animal Care and Use Committee approved protocol #71112, and in compliance with the USA Public Health Service Policy on Humane Care and Use of Laboratory Animals. Zebrafish were maintained under standard laboratory conditions [16]. *In situ* hybridizations were performed as described by Ahn et al (2002)[17]. Morpholino injections were performed as described by Nasevicius and Ekker (2000)[18]. 3.7 ng of Tbx5a morpholino (5’- CCTGTACGATGTCTACCGTGAGGC-3’)[17] and 5 ng of Tbx5b translation blocking morpholino (5’-GGATTCGCCATATTCCCGTCTGAGT-3’) [10] was injected per embryo.

### Tbx5b mutant generation

The gRNA sequence for CRISPR were generated using CHOPCHOP[19]. The specific sequence used was GAGCTGAGTTTGTAGTGGCG which targeted the first exon prior to the start of the TBOX domain. *Cas9* mRNA was generated from a plasmid from Jao et al[20]. gRNAs and *Cas9* mRNA were transcribed using methods previously described [21]. 100 ng of *Cas9* mRNA and 150 ng of Tbx5b gRNA were injected into F0 embryos. 30% showed phenotypes consistent with the Tbx5b mutant phenotype and died at 6 dpf. Surviving siblings were grown to adulthood, then outcrossed to *AB fish to generate stable F1 lines. These F1 lines were genotyped by tail clipping, amplifying the first two exons of *tbx5b* using the primers ztbx5bATGfwd[9] and 5’- CCATGCATAAATACCAGCCG-3’, TA cloning using pMD20 (Clontech) and then Sanger sequencing.

### RNA sequencing and analysis

*AB fish were injected with morpholinos at the single cell stage. For experiments where the eyes were removed, the dissection was performed using forceps prior to RNA extraction. RNA was extracted using TRIzol (Ambion) at either 18 or 21 hpf. 10 embryos were pooled for each library. Single-end 50 bp oligo dT cDNA libraries were generated and Illumina high throughput sequencing was performed. Differential gene expression was analyzed using 3 methods to identify significant differential gene expression. In all cases TopHat (v2.1.0) was used to align the RNA sequencing reads to the zebrafish genome (Zv9). Differential gene expression was identified using Cufflinks (v2.2.1) and Cuffdiff (v3.1.2); EdgeR[22]; or Cufflinks then the HOLT method [23]. Cuffdiff and Cufflinks analysis were performed using the Galaxy web platform[24]. The data discussed in this publication have been deposited in NCBI’s Gene Expression Omnibus [25] and are accessible through GEO Series accession number GSE115640 (https://www.ncbi.nlm.nih.gov/geo/query/acc.cgi?acc=GSE115640)

## Results

### *tbx5b* mutants phenocopy Tbx5b morpholino-injected embryos

**Fig 1:**
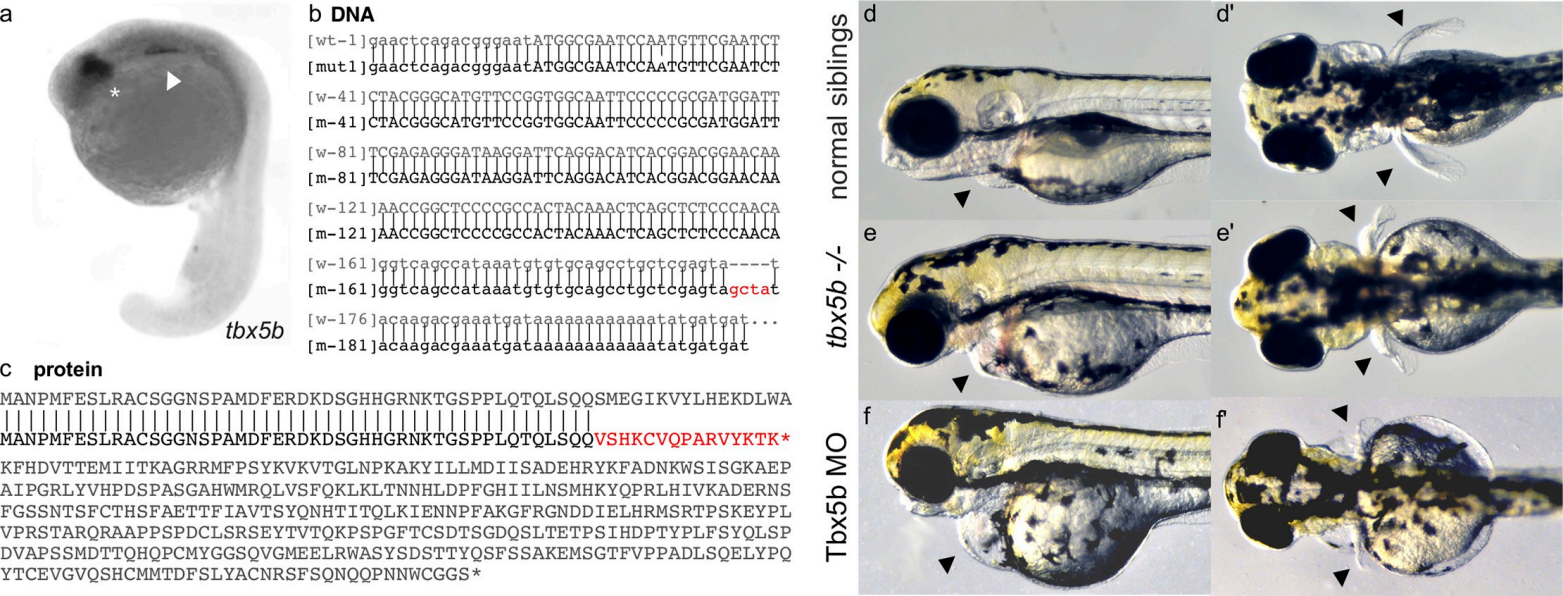
Tbx5b mutants phenocopy Tbx5b morphants. (a) *tbx5b* expression in the eyes (asterisk) and lateral plate mesoderm (arrow) of 21 hpf embryos. (b) The indel for the *tbx5b-2A* mutant (red) embryos is located in the beginning of the first intron. Uppercase letters are the protein coding sequence while lowercase letters are the untranslated regions and introns. (c) The mutation would produce a prematurely truncated protein with a frameshift resulting in incorrect amino acids starting at AA53 and a premature stop codon after 16 incorrect amino acids (red). (d-e) Embryos at 3 dpf. Arrows point to the heart and pectoral fins. Siblings from a Tbx5b in-cross display normal heart (d) and fin (d’) development while mutant siblings have affected hearts (e) and fins (e’) which phenocopy the defects seen in the heart (f) and fins (f’) of embryos injected with a Tbx5b morpholino.

Consistent with earlier studies[9,10,13], we report that *tbx5b* is expressed in the retina and in the anterior region of the lateral plate mesoderm of embryos during somitogenesis stages (Fig 1A). To further understand the effects of Tbx5b in the embryo, we used CRISPR Cas9 directed mutagenesis to create a Tbx5b mutant. The specific sequence used was GAGCTGAGTTTGTAGTGGCG which targeted the first exon prior to the start of the TBOX domain. 30% of the F0 injected embryos displayed phenotypes consistent with the characteristics exhibited by Tbx5b morphants, including hearts that failed to loop and small, malformed pectoral fins. Unaffected injected siblings were raised to adulthood and screened by in-crossing to identify mutant carriers. Once mutants had been identified by the phenotypes produced in the offspring, mutant F0 fish were outcrossed to produce stable F1 mutants. Sequencing identified a 4 base pair insertion (GCTA) in the first intron (Fig 1B). This mutation would result in a splicing error such that the first intron is not spliced from the protein, leading to a frameshift starting at amino acid 53 and a premature stop codon after 16 incorrect amino acids (Fig 1C). The protein would be truncated before the highly conserved TBOX (S1 Fig) and likely lead to a completely nonfunctional protein.

*Tbx5b-/-* mutants fully phenocopy the reported Tbx5b morphants[10,13]. *Tbx5b-/-* mutant embryos show the same heart defects displayed by Tbx5b morphant embryos. The heart fails to loop and a fluid filled edema forms surrounding the heart (Fig 1E and 1F). *Tbx5b-/-* mutants also exhibit similar fin defects to the Tbx5b morphants. Although the fin buds do initially form, they tend to be smaller[10] and eventually develop into malformed pectoral fins. At 3 dpf, when the pectoral fins of the wildtype embryo have extended posteriorly (Fig 1D’) both the Tbx5b mutants and the Tbx5b morphant embryos have significantly shorter, misshapen pectoral fins (Fig 1E’ and 1F’).

Additionally, there appear to be defects in tissues beyond the heart and the pectoral fin. The heads of Tbx5b-deficient and mutant embryos are reduced in size compared to wildtype siblings (Fig 1E and 1F). Overall head shape is truncated and rounder than wildtype embryos and the eyes appear to be slightly smaller. The jaws of affected embryos do not project as far anteriorly as do the jaws of similarly-stage wildtype embryos (S2 Fig). Additionally, unlike their wildtype siblings, the swim bladder of Tbx5b-deficient embryos does not inflate. By 6 days, both mutants and morphant embryos die, possibly as a result of the severe heart defects.

### RNA sequencing to identify differences between the Tbx5 paralogues

Whole embryo RNA sequencing was performed at both 18 hpf and 21 hpf. These time points were chosen as they represent the beginning of migration of the lateral plate mesoderm and a midpoint of lateral plate migration in the limb field[14]. Because both Tbx5a and Tbx5b morpholinos accurately recapitulate the mutant phenotypes and because the mutant phenotypes cannot be identified during the stages of limb field migration, morpholinos were used to create embryos deficient in either of the Tbx5 paralogues as well as in both. The use of morpholinos also allows for the assaying of single gene effects without paralogous gene compensation[26].

Preliminary results done on one replicate at each time point and analyzed using the Cuffdiff suite showed many genes that were differentially expressed were expressed in the eye (54 of the 153 genes with known tissue expression). To limit the differentially expressed targets to genes that may play a role in either heart or limb development, the eyes were removed before cDNA extraction for all subsequent experiments, although some comparisons do include the sequencing data performed using complete embryos (Preliminary Cuffdiff).

**Fig 2:**
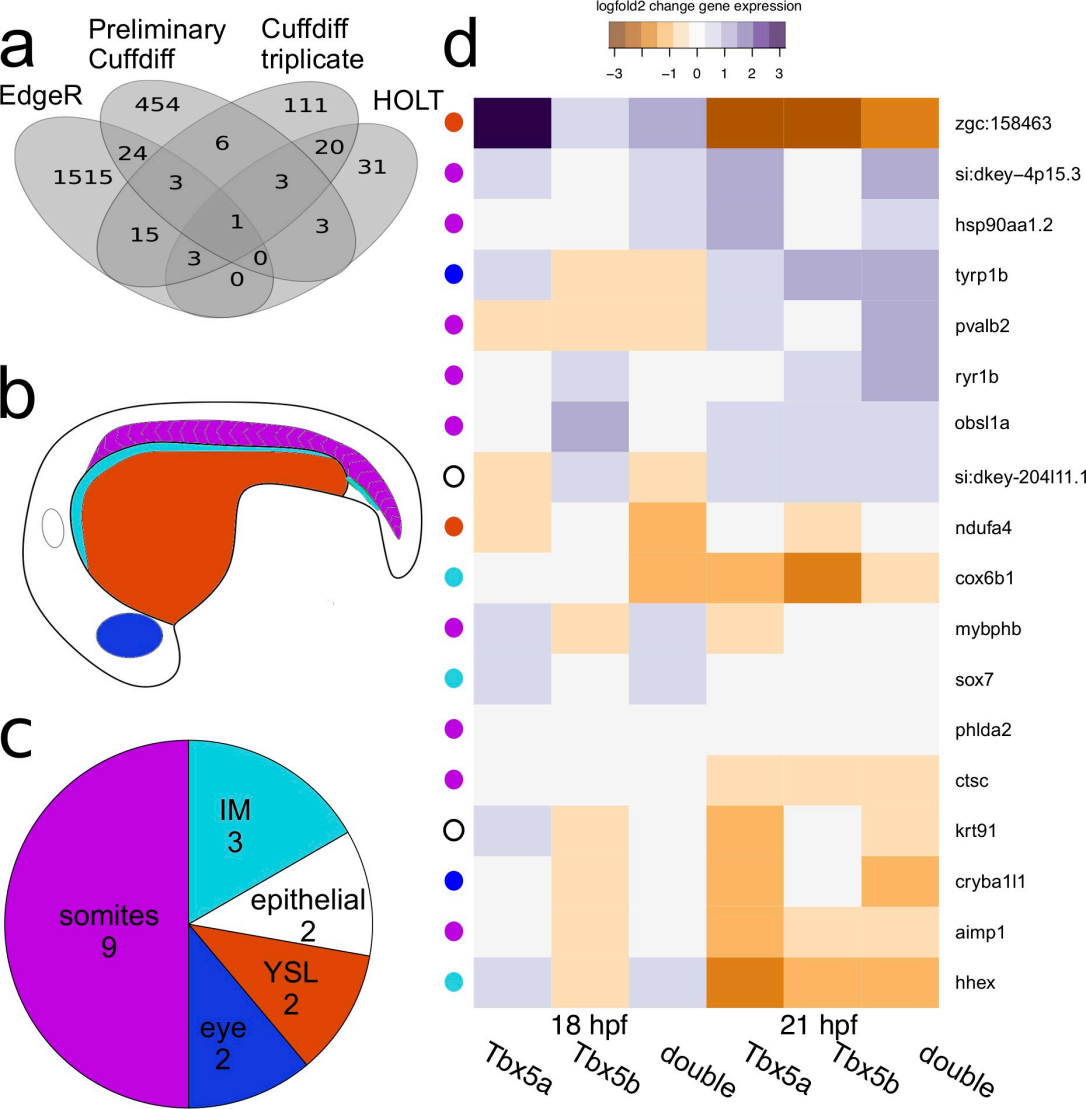
RNAsequencing data on Tbx5 paralogues. (a) Overlapping results of different methods of differential gene expression. (b) Schematic diagram of an embryo showing the different tissues where affected genes were expressed. (c) Breakdown of expression changes by location. (d) Differential gene expression in the confirmed genes. Colors are based on the fold change values generated in the Triplicate Cuffdiff differential expression. The colored dot to the left of the image corresponds with the main location of expression of the gene. White is epithelial expression, blue is eye expression, orange is expression in the Yolk Syncytial Layer (YSL), light blue is expression in the intermediate mesoderm (IM) and purple is somitic expression. The epithelial category encompasses peridermal and epidermal expression.

To create lists of differential gene expression for each genotype, expression levels were compared between deficient and wildtype embryos at the same time point. As described in the Methods Section, three different procedures for identifying gene expression were used in this study, which produced distinctive lists of differentially expressed genes (Fig 2A). The preliminary experiment produced 632 million reads and a list of 494 differentially expressed genes (detected using Cuffdiff), of which 454 were unique (Fig 2A). This final triplicate sequencing gave 309.4 million reads. When using Cuffdiff to produce differential gene expression lists, a list of 162 differentially expressed genes were identified. Of these genes, 111 were unique to this method of analysis (Fig 2A). Of note, only 13 genes were detected as differentially expressed between the preliminary and triplicate experiment, despite the fact that the only difference between them is number of replicates and removal of the eyes. The HOLT method identified the fewest number of differentially expressed genes, identifying 61 differentially expressed genes, of which 31 were unique to this method (Fig 2A). Conversely, EdgeR identified the greatest number of differentially expressed genes in this dataset, identifying 1561 differentially expressed genes, of which 1515 were unique to this dataset (Fig 2A). These results are consistent with other studies showing that different methods of identifying differential gene expression can produce relatively different lists of differential gene expression[27].

In order to select a smaller subset of these results for further screening, a list was created of genes that had been detected as differentially regulated by at least two methods of the analyses outlined above. This produced a list of 78 genes (Fig 2A, Full list is provided in S3 Fig) that were differentially expressed in Tbx5a-deficient, Tbx5b-deficient or doubly-deficient Tbx5a/b embryos (henceforth referred to as double-deficient embryos). However, not all expression changes were significant for each comparison for each gene. This is because the list was compiled of genes that had at least one significant differential expression change for any condition, not of genes that had significant differential expression changes for all conditions. When clustering the lists generated by differential gene expression for all conditions, we can see that the two separate time points first cluster together, suggesting that the largest difference in significant differential gene expression is temporal (S3 Fig). Next, for both time points, Tbx5a-deficient and double-deficient target genes cluster together, suggesting that in general the list of Tbx5a differentially regulated genes is more similar to the double-deficient differentially regulated genes list than it is to the Tbx5b list.

### Candidate gene identification and in situ hybridization

To understand spatiotemporal information about the results of this whole-embryo RNA-sequencing experiment, *in situ* hybridizations were performed for the 78 genes on the combined list. This provided information about the tissue in which a target gene was located in and if there were any detectable changes in expression levels or patterning in Tbx5a-deficient, Tbx5b-deficient, or double-deficient embryos at different time points.

Of the 78 candidate genes, 18 were confirmed to have specific changes in expression that was detectable by *in situ* expression changes. These genes were expressed in a variety of tissues (Fig 2B and 2C). Nine genes were identified as being differentially expressed in the somites, three genes were identified as being expressed in the intermediate mesoderm, two genes were identified as being expressed in the yolk syncytial layer, two genes were identified as being differentially expressed in the eye, one gene was identified as being differentially expressed in the periderm, and one gene was identified as being differentially expressed in epidermal tissue (a full list is provided in Fig 2D).

### Tbx5 paralogues show changes in expression in the vasculature

**Fig 3:**
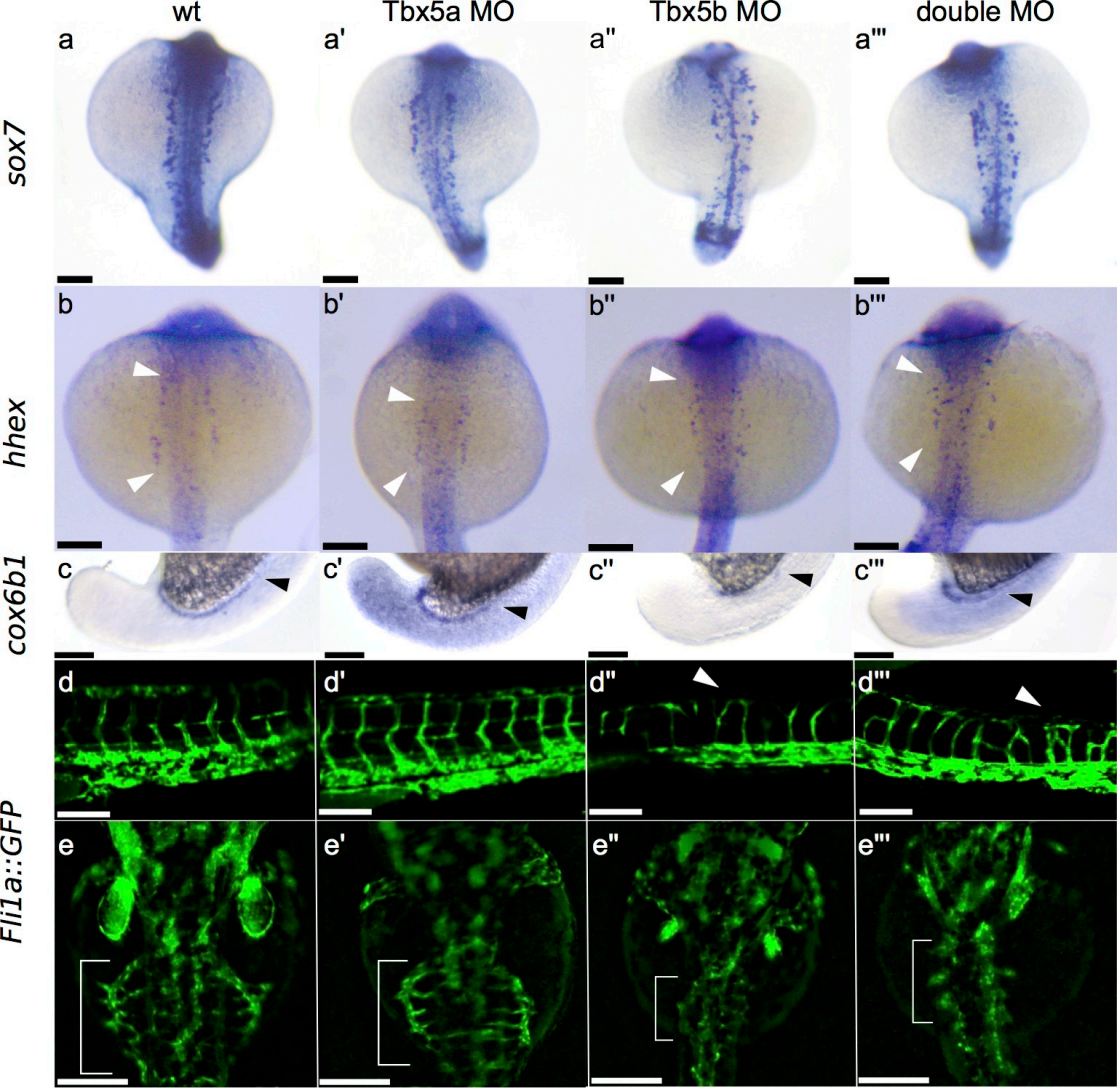
Effects of Tbx5 paralogues on vasculature. (a-a”’) Dorsal view of *sox7* expression at 18 hpf showing misexpression in Tbx5a-deficient embryos (a’), Tbx5b-deficient embryos (a”) and double-deficient embryos (a”’) compared to normal wildtype expression (a). (b-b”’) Dorsal view of *hhex* expression at 21 hpf displaying mild misexpression in Tbx5a-deficient embryos (b’) and more severe misexpression in Tbx5b-deficient embryos (b”) and double-deficient embryos (b”’) compared to wildtype (b). (c-c”’) Lateral view of *cox6b1* expression at 18 hpf in the developing pronephric duct. Arrowheads mark the anterior limit of expression. Expanded expression is present in the surrounding tissue of Tbx5a-deficient (c’) and double-deficient (c”’) embryos, while expression is decreased throughout the embryo in Tbx5b-deficient embryos (c”). (d-d”’) Lateral view of the *Tg(fli1a*::*EGFP)y1* line displays increased branching in the intersomitic vessels of both Tbx5b-deficient (d”) and double-deficient embryos (d”’) (see arrowheads) compared to either wildtype (d) or Tbx5a-deficient embryos (d’). (e-e”’) Dorsal view of the *Tg(fli1a*::*EGFP)y1* line. The subintestinal vessels are marked with a bracket in 3 dpf embryos. There is a decrease in size of the subintestinal vasculature in Tbx5b-deficient (e”) and double-deficient (e”’) embryos compared to either wildtype (e) or Tbx5a-deficient embryos (e’). Scale bars are 100 μm.

Three of the genes were expressed in the intermediate mesoderm, a tissue which lies immediately medial to the LPM and differentiates into blood/endothelial precursors and the pronephric tubules. Both *sox7* and *hhex* are expressed primarily in endothelial precursors[28,29], while *cox6b1* is expressed in the developing pronephros. Vasculature and endothelial precursors originate in the intermediate mesoderm and then migrate to the midline during somitogenesis[28]. *Sox7* in particular shows a distinctive change in both Tbx5b-deficient and double-deficient embryos, which appears to correspond with a disrupted or disorganized migration of angioblasts migrating to the dorsal aorta (Fig 3A and 3A”’).

Of the two genes mis-regulated in the vasculature, *sox7* showed the most striking effect. In wildtype embryos, the vasculature precursors are arranged in a regular fashion on both sides of the midline at 18 hpf (Fig 3A). In Tbx5a-deficient embryos, there is an increased disorganization of these precursor cells (Fig 3A’). Both Tbx5b-deficient and double-deficient embryos show a strong decrease in organization of these cells, as well as an apparent decrease in the number of expressing cells present (Fig 3A” and 3A”’).

The gene *hhex* is misexpressed at 21 hpf by *in situ* hybridization. In wildtype embryos, *hhex* is expressed in vasculature precursors (Fig 3B) as well as in the intermediate cell mass located post yolk-extension which will contribute to either the pronephros or blood/endothelium. The wildtype expression is present in two orderly stripes on both sides of the embryo (see arrowheads marking the extent of the expression). In Tbx5a-deficient embryos the extent of this expression is slightly decreased (see white arrowheads marking the extent of expression) compared to wildtype embryos (Fig 3B’), however the expression is still located in two orderly stripes. However, both Tbx5b-deficient and double-deficient embryos show a scattered expression of these cells, including expression closer to the midline than in either wildtype or Tbx5a-deficient embryos (Fig 3B” and 3B”’). This is consistent with the *sox7* data suggesting that there is both a strong downregulation and mis-expression of vasculature precursors in Tbx5b-deficient and double-deficient embryos.

The gene *cox6b1* is expressed in the developing pronephros (Fig 3C, arrowhead marks the anterior extent). In Tbx5a-deficient embryos, there is ectopic *in situ* expression present in the surrounding tissue of the embryo, as well as an increased level of expression in the pronephros (Fig 3C’). However, Tbx5b-deficient embryos show a decrease in *in situ* levels of expression, with only faint levels of *cox6b1* expression remaining in the pronephros (Fig 3C”). This downregulation is consistent with the significant differential gene expression data, which found that *cox6b1* was significantly downregulated in Tbx5b-deficient embryos compared to wildtype embryos at 18 hpf (preliminary cuffdiff data). Interestingly, *cox6b1* expression in the double-deficient embryos is also expanded and appears more similar to Tbx5a-deficient embryos (Fig 3C”’). The RNA sequencing data only detected a significant change in differential gene expression at 18hpf in Tbx5b-deficient embryo in both the preliminary and the triplicate experiments.

To test if there are any underlying defects in the vasculature, we used the *Tg*(*fli1a*::*EGFP)y1* transgenic line to examine the structure of the vasculature[30]. The intersomitic vessels (ISV) of normal embryos show a clear “V shaped” pattern as these vessels form in between the somites (Fig 3C). Notably, these vessels are patterned prior to fluid flow, so the patterning of the ISV should not be affected by possible changes in fluid flow due to the Tbx5-deficient paralogues’ defective hearts. Tbx5a-deficient embryos exhibit normal branching in the ISV (Fig 3D’). However, Tbx5b-deficient embryos display minor defects in the ISV with additional ectopic branching (arrowhead, Fig 3D”). Double-deficient embryos show an even stronger defect in the structure of the ISV, exhibiting an increase in ectopic branching points (arrowhead, Fig 3D”’).

We also examined the subintestinal vessels (SIV) of embryos at 3 dpf. In wildtype embryos the SIV spreads out over the yolk with small vessels leading back to the midline along the length of the SIV (Fig 3E, in brackets). Tbx5a-deficient embryos in general display an overall SIV morphology that is relatively normal sized and shaped (Fig 3E’). However, the size of the SIV is largely decreased in Tbx5b-deficient embryos (Fig 3E”) and almost completely missing in double-deficient embryos (Fig 3E”’). This suggests that the Tbx5 paralogues may be playing a role in vasculogenesis.

### Somitic genes are the largest category of differentially expressed genes

The largest category of differentially expressed genes were those expressed in the somites. Although all of these genes were misexpressed in the affected embryos, three of these genes, *hsp90aa1*.*2*, *mybphb*, and *phlda2*, showed misexpression patterns most strongly at 18 hpf (S4 Fig). Six genes, *obsl1a*, *pvalb2*, *si*:*dkey-4p15*.*3*, *ctsc*, *aimp1*, and *ryr1b*, showed misexpression patterns most strongly at 21 hpf (S4 Fig).

**Fig 4:**
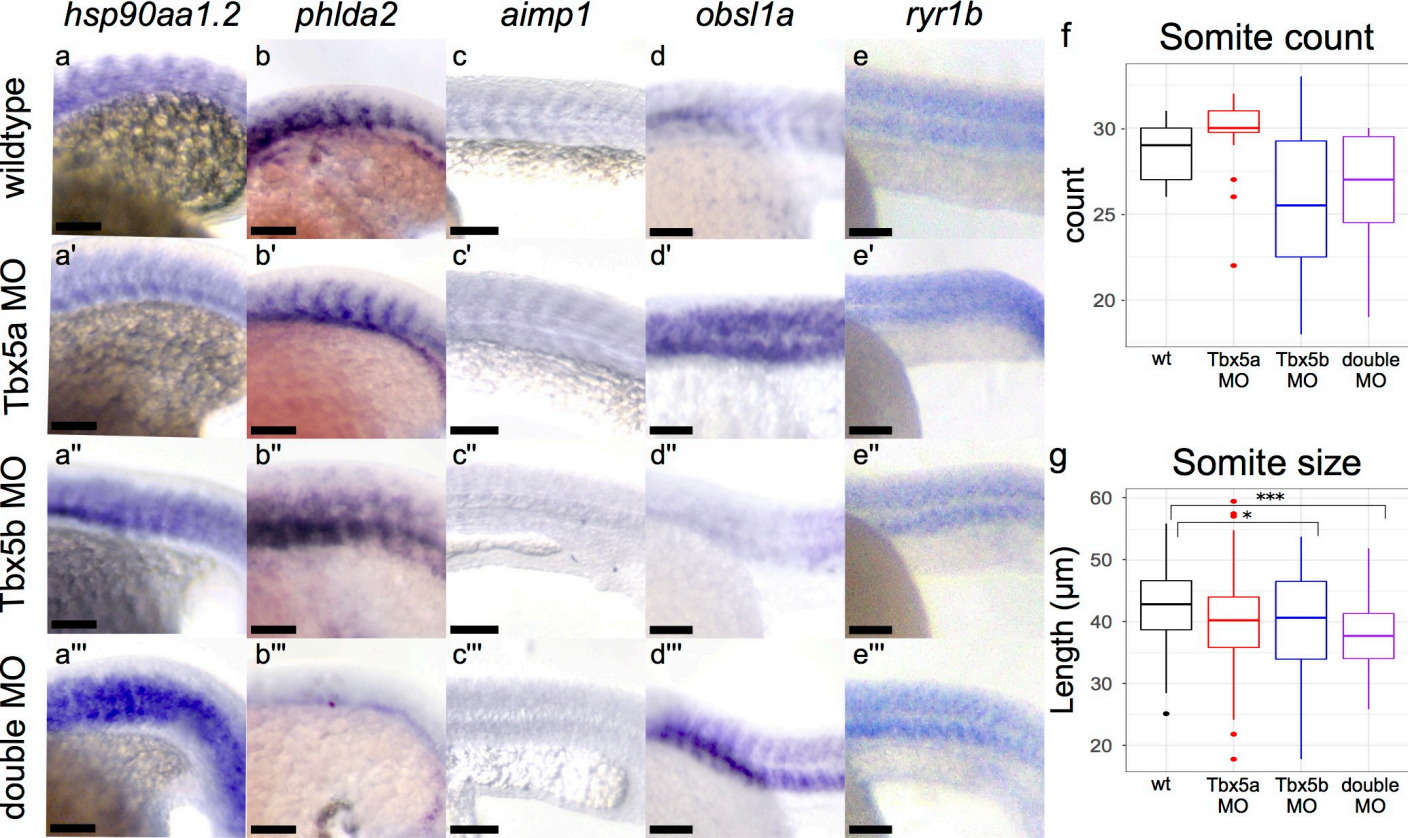
Effects of Tbx5 Paralogues on the Somites. All views are lateral, see text for details. *hsp90aa1*.*2* expression at 18 somite stage is expanded in Tbx5b-deficient (a”) and double-deficient (a”’) embryos compared to wildtype (a). *phlda2* expression at 18 hpf is consistent in the somites in wildtype (b), Tbx5a-deficient (b’) and Tbx5b-deficient (b”) embryos, but mostly absent in double-deficient embryos(b”’). Expression of *aimp1* at the 21 hpf stage is decreased in Tbx5a-deficient (c’), Tbx5b-deficient (c”) and double-deficient (c”’) embryos compared to wildtype embryos (c). In 21 hpf embryos, *obsl1a* expression is increased in the somites of Tbx5a-deficient (d’) and double-deficient (d”’) embryos compared to wildtype (d) embryos. Tbx5b-deficient embryos show a decrease in expression in the trunk somites (d”) but an increase in the more posterior somites (S4F” Fig) compared to wildtype embryos. Expression of *ryr1b* is increased slightly in the Tbx5a-deficient (e’) embryos compared to wildtype embryos (e) and most strongly in the double-deficient embryos especially in the ventral somites(e”’). Expression of *ryr1b* in the Tbx5b-deficient embryos (e”’) is more similar to wildtype than to Tbx5a-deficient embryos. Scale bar is 100 μm. (f) Somite count of embryos at 25 hpf. Tbx5a-deficient (n = 16), Tbx5b-deficient (n = 12), and double-deficient (n = 15) showed no significant difference in somite count compared to wildtype (n = 15). (g) Somite length measured along the AP axis of wt (n = 104), Tbx5a-deficient (n = 110), Tbx5b-deficient (n = 66) and double-deficient (n = 96). Both Tbx5b-deficient and double-deficient embryos showed a significant decrease in somite size compared to wt embryos. (g-f) significance was tested using ANOVA and Tukey HSD test, *p<0.05, ***p<0.0001.

The gene *hsp90aa1*.*2* is a heat shock protein and upregulated in all morphant conditions at 18 hpf (Fig 4A and 4A”’). Wildtype embryos have *hsp90aa1*.*2* expression clearly expressed within somites (Fig 4A). In Tbx5a-deficient embryos, *hsp90aa1*.*2* expression is upregulated especially in the anterior somites (Fig 4A’). In Tbx5b-deficient embryos, *hsp90aa1*.*2* is also upregulated in the anterior somites but additionally appears to be misexpressed throughout the entire somite structure as well, as compared to either the wildtype or the Tbx5a-deficient embryos (Fig 4A”) in which boundaries of expression between somites can be seen more clearly. Double-deficient embryos exhibit even stronger staining throughout the embryo, with staining no longer limited between somitic boundaries (Fig 4A”’). The gene *phlda2* is also expressed in the somites at 18 hpf, as well as in more anterior head mesenchyme (Fig 4B, S4A Fig). Both the *in situs* and the RNA sequencing data show a decrease in embryo-wide expression of *phlda2* in single-deficient embryos at 18 hpf compared to wildtype embryos (S4A’ and S4A” Fig), although the levels of expression specifically in the somites appear consistent for both Tbx5a-deficient and Tbx5b-deficient embryos. However, when we examine the whole body *in situ* images, we can see that expression in the anterior regions of the embryo is specifically decreased compared to either Tbx5a-deficient or Tbx5b-deficient embryos which may explain the RNA sequencing results (S4A and S4A” Fig). However, double-deficient embryos show a strong loss of expression both in the somites and throughout the whole embryo (Fig 4B”’, S4A”’ Fig).

The gene *aimp1* is expressed in all somites at 21 hpf (Fig 4C, S4H Fig). Consistent with the whole-embryo differential expression data, the level of expression appears to be decreased in all morphant embryos at 21 hpf, most notably in the Tbx5b-deficient and double-deficient embryos (Fig 4C’ and 4C”’, S4H’ and S4H”’ Fig). Expression for *obsl1a* expression at 21 hpf resides both in the eye and in the somites (Fig 4D, S4F Fig). Consistent with the RNA sequencing differential expression levels, *obsl1a* expression appears to be upregulated in Tbx5a-deficient embryos in the somites at 21 hpf, although expression in the eye appears to be lost in these embryos (Fig 4B’, S4F’ Fig). Tbx5b-deficient embryos show decreased expression of *obsl1a* in the trunk somites but an increased expression level compared to wildtype embryos in the more posterior somites (Fig 4D”, S4F” Fig). This is consistent with the RNA sequencing data as it shows a less strong upregulation than Tbx5a-deficient embryos, which could be due to the uneven upregulation throughout the body of the embryo (Fig 4D”, S4F” Fig). Double-deficient embryos show a general increase in expression of *obsl1a* throughout all the somites, although this upregulation appears to be stronger in the ventral half of the somites than in the dorsal half (Fig 4D”’). At 21 hpf, *ryr1b* is expressed broadly throughout the somitic mesoderm (Fig 4E, S4I Fig). The whole embryo RNA sequencing data shows a trend towards upregulation in all morphant conditions, although this change is only significant in the double-deficient compared to wildtype condition. This is consistent with the *in situ* expression data, in which an increase in expression of *ryr1b* in the double-deficient embryos (most strongly in the dorsal somites) is observed (Fig 4E”’, S4I”’ Fig). There is also a potentially a slight increase in expression in the Tbx5a-deficient embryos compared to wildtype, especially in the more posterior somites (Fig 4E’, S4I’ Fig). Expression of *ryr1b* in the Tbx5b-deficient embryos (e”’) is more similar to wildtype than to Tbx5a-deficient embryos. Since the RNA sequencing data showed no significant change in differential gene expression in the Tbx5b-deficient embryo, this *in situ* data is consistent with the RNA sequencing data.

However, the remaining expression by *in situ* hybridization for the somite specific genes does not perfectly correspond with the whole embryo differential expression data. The following genes are either partially consistent or inconsistent between the RNA sequencing data and the *in situ* hybridization data. These genes may represent false positives or they may represent the limitations of using *in situ* hybridizations as a quantitative measurement. At 18 hpf, *mybphb* is expressed only in the dorsal half of the somites in wildtype embryos. It is upregulated in Tbx5a-deficient embryos, with the upregulation most strongly seen in the somites but also in the head of the embryo (S4B and S4B’ Fig). The Tbx5b-deficient embryos show an expansion of expression in the somites compared to wildtype, with expression detectable in both the dorsal and ventral half of the somites, with a loss of expression in the head (S4B” Fig). The double-deficient embryos show an increase in levels of expression of *mybphb* in the anterior somites, but a decrease in expression of *mybphb* in the posterior somites and the head, making it difficult to compare levels of expression with wildtype (S4B”’ Fig). This is mismatched with the RNA sequencing data which shows significant downregulation only in the Tbx5a-deficient embryos compared to wildtype at this time. The gene *pvalb2a* is upregulated in all morphant conditions at 21 hpf by RNA sequencing, although it is not noticeably upregulated in the Tbx5a-deficient embryos by *in situ* (S4D and S4D”’ Fig). The *in situ* expression changes for *pvalb2a* show a change in pattern at both the Tbx5b-deficient and double-deficient embryos, with expression appearing to be stronger, but limited to more anterior somites when compared to wildtype, which makes it difficult to conclusively determine if the levels of *pvalb2a* are differentially regulated on a whole-embryo level. Expression levels of *si*:*dkey-4p15*.*3* appear to be upregulated and expanded throughout the somites in all deficient cases by *in situ* (S4E and S4E”’ Fig), however it is only significantly upregulated in the RNA sequencing data in the Tbx5a-deficient condition. Furthermore, there is a discrepancy in *si*:*dkey-4p15*.*3* levels in Tbx5b-deficient embryos which appear to be differentially downregulated by the EdgeR comparison at 21 hpf, despite apparent increase in expression as seen by *in situ* (S4E”’ Fig). The gene *ctsc* is expressed specifically at the middle of the somite and found to be significantly downregulated by RNA sequencing in both the Tbx5a-deficient and Tbx5b-deficient embryos at 21 hpf; in contrast the *in situ* expression in these embryos appears to be expanded (S4G and S4G”’ Fig).

Despite the large number of somite-specific genes differentially expressed in this study, gross somite morphology appears similar in wildtype and Tbx5-deficient embryos (Fig 1F), as well as in Tbx5a mutants or Tbx5a-deficient embryos[12,17]. To test if the size or number of somites was different, we measured somite size and counted somite number in embryos deficient in the Tbx5 paralogues at 25 hpf, one hour after the end of somitogenesis[31]. There were no significant changes in somite count in the Tbx5-deficient embryos compared to wildtype (Fig 4F). There was a significant decrease in length of the somites in both the Tbx5b-deficient and double-deficient embryos compared to wildtype, but no decrease in somite length in Tbx5a-deficient embryos compared to wildtype (Fig 4G).

### Changes in other tissues

The retina is another site of expression of both Tbx5 paralogues. Both *cryba1l1* and *tyrp1b* are primarily expressed in the eye (*cryba1l1*, *tyrp1b*) and show the largest differential expression changes there. *The gene cryba1l1* is expressed in the wildtype eye at 21 hpf (S5A Fig). In Tbx5a- deficient embryos, *cryba1l1* expression is reduced to a smaller region of the eye (arrowhead), consistent with the significant downregulation at this phase (S5A’ Fig). The expression also appears to be decreased in both Tbx5b-deficient and double-deficient embryos, despite no significant change in gene expression as detected by whole embryo RNA sequencing (S5A” and S5A”’ Fig). The gene *tyrp1b* is expressed in the dorsal-posterior part of the developing eye at 21 hpf, and is expressed at higher levels in all Tbx5 deficient embryos, consistent with the whole embryo RNA sequencing data (S5B and S5B”’ Fig). Notably, *tyrp1b* expression in the Tbx5a-deficient embryos appears stronger in the eye than in the wildtype embryos (S5B and S5B’ Fig). While Tbx5b-deficient and double-deficient embryos show a strong increase in staining intensity, the expression is reduced to a smaller portion of the eye, especially in the Tbx5b-deficient embryos (expression boundaries marked by arrowheads, S5B” and S5B”’ Fig).

Two genes were expressed in the yolk syncytial layer (YSL), *zgc*:*158643* and *ndufa4*. Expression of *ndufa4* was increased in the double-deficient embryos at 18 hpf (S5C”’ Fig); however the triplicate cuffdiff data found that *ndufa4* was significantly downregulated in the double-deficient compared to wildtype (Fig 2D). The gene *zgc*:*158643* is expressed in an expression pattern that appears to mimic the position of the yolk granule cells, as well as in the tail (S5D Fig). Expression of *zgc*:*158643* in Tbx5a-deficient embryos appears to be expanded along the yolk, while in both Tbx5b-deficient and double-deficient embryos, expression is expanded to cover the entire yolk, as well as expanded in the tail and body of the embryo (S5D’ and S5D”’ Fig).

Two genes, *si*:*dkey-204l11*.*1* and *krt91*, were differentially expressed in the periderm or epidermis of the embryo. The gene *si*:*dkey-204l11*.*1* appears to be downregulated in Tbx5a-deficient and double-deficient embryos, consistent with the differential expression detected by RNA sequencing (S5E and S5E”’ Fig). *in situ* expression of *krt91* appears to be greatly downregulated in only the double-deficient embryos at 21 hpf, although the RNA sequencing data show a significant change in differential gene expression in Tbx5a-deficient embryos compared to wildtype (S5F and S5F”’ Fig).

## Discussion

This study reports on whole embryo transcriptional changes in response to loss of *tbx5a* and *tbx5b* gene functions. As part of this analysis, a Tbx5b mutant was engineered and briefly described to show that injections of morpholinos directed to *tbx5b* will phenocopy a null mutation. Both the mutants and the morphants display similar heart defects and have small, misshapen pectoral fins, Notably, Tx5b mutants and morphants both display similar changes in the overall morphology of the head and jaw; these phenotypes, which are not observed in embryos deficient in the paralogous Tbx5a gene, will be the focus of a future study.

An RNA-sequencing analysis comparing the transcriptomes of Tbx5 deficient embryos identified 78 genes with differential gene expression that was corroborated by *in situ* hybridization. Most of these identified targets were not expressed in the lateral plate mesoderm, but were expressed in surrounding tissues. Two of the larger classes of differentially expressed genes were found to be specific to either the vasculature or somitic tissues. Notably, the two gene expression changes we found in the vasculature produced later corresponding morphological defects in Tbx5b-deficient and double-deficient embryos. There were also nine genes misexpressed in the somites of Tbx5 deficient embryos, suggesting possible changes in somitic cell-derived functions, although overall patterning and development of the early somites in Tbx5b-deficient embryos display only minor differences compared to wild-type siblings.

Tbx5b appears to be regulating vasculature formation, with possible contribution from Tbx5a as Tbx5a-deficient embryos do not display a vasculature phenotype, but double-deficient embryos exhibit a worse phenotype than single Tbx5b knockdown. The observation that Tbx5 paralogues affect vasculature development is novel. Interestingly, we observed both an increase of branching and vasculature formation in the ISV and a decrease in branching and vasculature formation in the SIV (Fig 3D–3E”’). How can we reconcile these opposite effects in the vasculature? This may be due to the different methods of formation of these tissues. The SIV is a flow-dependent tissue—it’s growth and patterning is at least partially dependent on the existence of fluid flow[29]. Therefore, the decrease in SIV size and branching could be partially explained by the loss or decrease of fluid flow due to the heart defects in Tbx5b-deficient embryos. Although this does not fully explain why Tbx5a-deficient embryos appear to have normal SIV, it could indicate that the similar Tbx5b-deficient heart phenotype actually results in a less effective circulation than the Tbx5a-deficient hearts. In comparison, the ISV are patterned prior to the establishment of fluid flow in those vessels, so ISV should not be affected by pressure differences due to the heart defects. The defects observed in ISV may therefore be due to more direct functional effects of loss of the Tbx5 genes.

The decreases in *sox7* expression are particularly noteworthy because it suggests interaction between Tbx5 genes with the VEGF/Notch signaling pathways that are known to control vasculature branching. Patterning of the vasculature depends partially on a balance between VEGF and Notch signaling[32]. Overexpression of VEGF signaling results in ectopic branching while overexpression of Notch signaling leads to a decrease in branching. Extra branching in the ISV is also seen in double *sox7*/*sox18* knockdowns, although not in either single knockdown or the *sox7* mutant [29,33]. Furthermore, s*ox7* expression is dependent on Shh and VEGF signaling but is upstream of Notch signaling. [34] This increased branching is consistent with a reduction of Notch signaling, which would be consistent with a decrease in *sox7* expression seen in Tbx5b-deficient and double-deficient embryos.

As the ISVs are patterned between the somites, there could also be a connection between the differential target gene expression in the somites and the mispatterning in the vasculature. For instance, *hsp90aa1*.*2* has been found to promote both VEGF and Notch signaling via HIF1 as an intermediary[35]. The gene *hsp90aa1*.*2* also has three TBOX consensus sites located in its introns. It is upregulated in both the Tbx5b-deficient and double-deficient embryos compared to wildtype, which correlates with the increased branching seen in the vasculature. Another target in the somites is *aimp1*, which is secreted and inhibits angiogenesis by affecting endothelial migration and adhesion[36]. Since *aimp1* appears to be downregulated in both the Tbx5b-deficient and double morphants, this downregulation of *aimp1* at the somite boundaries could potentially be partially responsible for the increased branching of the ISV.

Interestingly, in regards to the genes expressed in the vasculature, Tbx5b-deficient embryos and double-deficient embryos exhibited more similar phenotypes compared to Tbx5a-deficient embryos (Fig 3A–3B”’). However for the pronephros gene *cox6b1*, the opposite was observed, i.e. the Tbx5a-deficient phenotype was more similar to the double-deficient (Fig 3C–3C”’). This may indicate that the *tbx5* paralogues differentially affect separate components of the intermediate mesoderm through downstream signaling pathways.

How can Tbx5b be affecting tissues that it does not appear to be expressed in? For instance, although expression in the limb can be seen by 36 hpf[10], mutant phenotypes appear far earlier, with noticeable delays and decreased size of the limb bud visible as early as 30 hpf. One possible answer is that, perhaps at least in the limb, Tbx5b is present prior to 36 hpf, but at a level lower than that detectable by *in situ* hybridizations. However, the RNA sequencing also identified effects of Tbx5b in many additional tissues, including the vasculature and the somites. Another explanation for how Tbx5b is affecting tissues that it is not expressed in is that Tbx5 paralogues may be acting non-cell autonomously. This has been previously shown in mice, where TBX5 knockdown was shown to have a non-cell-autonomous action in muscle patterning in the forelimb [37]. However, RNA sequencing alone cannot determine whether a transcription factor is directly or indirectly affecting the expression levels of a specific gene, as the change in expression levels could also be due to other genes affected elsewhere in the transcriptional network.

Furthermore, this data appears to show that both Tbx5 paralogues are involved in a wide range of transcriptional networks that extend beyond the tissue that these genes are expressed in. This work has allowed for an examination of the embryo-wide changes in gene expression and characterization of the environment through which the cells of the lateral plate mesoderm are migrating. The fact that this screen did not recover any significant network of genes that appears to be exclusively expressed in the lateral plate mesoderm could be due to the relative tissue size. The lateral plate mesoderm is a relatively small tissue at 18 and 21 hpf, consisting of approximately 300 cells per embryo. Therefore, it is perhaps not too surprising that so many genes recovered in this experiment were misexpressed in the somites, one of the largest components of embryonic mesoderm. Another possibility is that of the 78 genes found to be differentially expressed via RNA sequencing, many of them may have been differentially regulated in the LPM, but not at a level or pattern detectable by *in situ* hybridization.

One may question why there have not been somite or vasculature defects previously associated with Holt-Oram syndrome patients or in Tbx5 mutant mice. One possible reason is that this study allows a higher level of resolution than that available, particularly in humans. These defects are subtle defects that may not be assayed as easily in other animals. For instance, although the intersomitic vasculature was mispatterned in the Tbx5 double-deficient embryos, it was still present and appeared functional. Furthermore, because these are subtle phenotypes, they may not be present in Holt-Oram patients, who still have one functioning copy of Tbx5. Additionally, zebrafish also are able to reach a more mature stage of development when lacking the Tbx5 paralogues than mice embryos, as mice embryos lacking *Tbx5* stall in development at embryonic day 9.5 due to heart defects[7], which may prevent these phenotypes from being detected.

In conclusion, this study summarizes an RNA sequencing experiment performed on the Tbx5a and Tbx5b paralogues in zebrafish. It found that gene expression levels were affected in many different tissues, notably the vasculature and the somites. These changes in gene expression are also reflected morphologically in the affected tissues, with changes occurring both in the vasculature and the somites of Tbx5a- and Tbx5b-deficient embryos.

## Supporting information

S1 Fig Protein alignments of Tbx5 genes. This figure shows the protein conservation between *Tbx5* in chick, human, mouse and zebrafish. The TBOX is highlighted in yellow. The *tbx5b-2a* mutation produces a protein truncation prior to the start of the TBOX domain.
(TIFF)

S2 Fig Alcian blue staining in the jaw at 6 dpf. Wildtype siblings show a more protruding lower law that extends beyond the eye as can be seen both from a lateral (a) and ventral (a’) view. Affected *tbx5b* -/- mutant embryos have a rounder head and a jaw that does not protrude beyond the eyes, as seen from both a lateral (b) and ventral (b’) view. Scalebar is 100 μm.
(TIFF)

S3 Fig Differential expression of all overlapping genes. Expression changes compared to wildtype embryos at each time point for all genes that were identified using 2 or more differential gene expression methods. Differential gene expression values were determined using the Cuffdiff data for this figure.
(TIFF)

S4 Fig Complete somite differential expression. *In situ* hybridization of all genes differentially expressed in the somites. (a-a”’) At 18hpf, *phlda2* is upregulated in Tbx5a-deficient (a’) and Tbx5b-deficient (a”) embryos compared to wildtype embryos (a’), but downregulated in the double-deficient embryos (a”’). At 18hpf, *mybphb* shows upregulation in Tbx5a-deficent (b’) and double-deficient (b”’) embryos compared to wildtype embryos (b). At 18hpf, *hsp90aa1*.*2* is upregulated in Tbx5a-deficient (c’), Tbx5b-deficient (c”) and double-deficient (c”’) embryos compared to wildtype (c) embryos, especially in the anterior somites. At 21 hpf, *pvalb2* expression is increased in Tbx5b-deficient (d”) and double-deficient (d”’) embryos compared to wildtype (d) but not Tbx5a-deficient embryos (d’). At 21 hpf, *si*:*dkey-4p*:*15*.*3* expression is increased in Tbx5a-deficient (e’), Tbx5b-deficient (e”) and double-deficient (e”’) embryos. At 21 hpf, *obsl1a* expression is upregulated in Tbx5a-deficient (f’), Tbx5b-deficient (f”) and double-deficient (f”’) embryos compared to wildtype (f) embryos. At 21 hpf, *ctsc* expression is expanded in Tbx5a-deficient (g’) and Tbx5b-deficient (g”) embryos compared to wildtype (g) embryos. At 21 hpf, *aimp1* expression is decreased in Tbx5a-deficient (h’), Tbx5b-deficient (h”) and double-deficient (h”’) embryos compared to wildtype embryos (h). At 21 hpf, *ryr1b* expression is increased in both Tbx5a-deficient (i’) and double-deficient (i”’) embryos compared to wildtype (i) embryos. (j-k) Comparison of length between Tbx5b mutant embryos and siblings, n = 10, measurements in μm. (j) Tbx5b-deficient embryos at 3 dpf are significantly shorter than their siblings. (k) Somite size is not significantly different at 3dpf between Tbx5b-deficient embryos and their wildtype siblings. Since somite size varies along the AP axis, measurements were taken of the more anterior somites only. (l) At 25 hpf, there is a significant difference in somite number between wildtype and Tbx5b morpholino injected embryos. Scale bar is 100 μm.
(TIFF)

S5 Fig Differential expression in other tissues. All views are lateral. At 21 hpf, *cryba1l1* is downregulated in Tbx5a-deficient (a’), Tbx5b-deficient (a”), and double-deficient (a”’) embryos compared to wildtype (a) embryos in the eye. Arrowheads mark the expression. At 21 hpf, *tyrp1b* expression is expressed at higher levels in Tbx5a-deficient(b’), Tbx5b-deficient (b”) and double-deficient (b”’) eyes compared to wildtype (b) eyes. Arrowheads mark the limits of the expression domain. At 18 hpf, *ndufa4* is upregulated in the double-deficient embryo (c”’) compared to wildtype (c) embryos. Expression of *zgc158642* at 21 hpf is upregulated in Tbx5b-deficient (d”) and double-deficient (d”’) embryos compared to wildtype embryos (d). At 18 hpf, *si-dkey-204l11*.*1* expression is downregulated in Tbx5a-deficient (e’) and double-deficient (e”’) embryos compared to wildtype (e) embryos. At 21 hpf, expression of *krt91* is downregulated in double-deficient embryos (f”’) compared to wildtype embryos (f). Note f” has normal tail length, but it is bent out of focus of this image. Scale bars are 100 μm.
(TIFF)
