# Supplementary figures and images for: A transcriptomics analysis of the Tbx5 paralogues in zebrafish
Source: PLoS One. 2018 Dec 10;13(12):e0208766. doi: 10.1371/journal.pone.0208766 (PMC6287840; doi:10.1371/journal.pone.0208766)

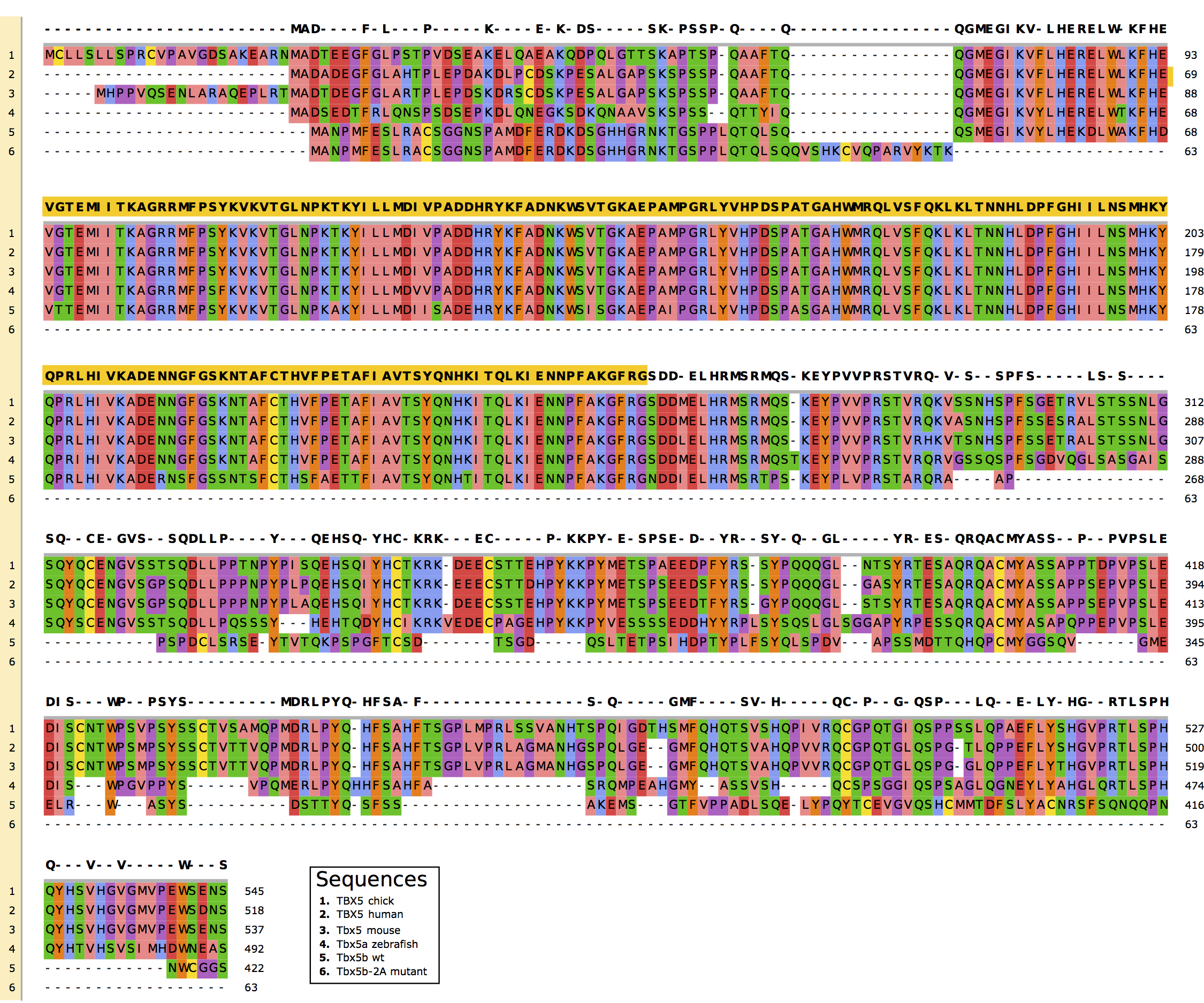

Supplement: S1 Fig — This figure shows the protein conservation between Tbx5 in chick, human, mouse and zebrafish. The TBOX is highlighted in yellow. The tbx5b-2a mutation produces a protein truncation prior to the start of the TBOX domain. (TIFF) [file pone.0208766.s001.tiff]

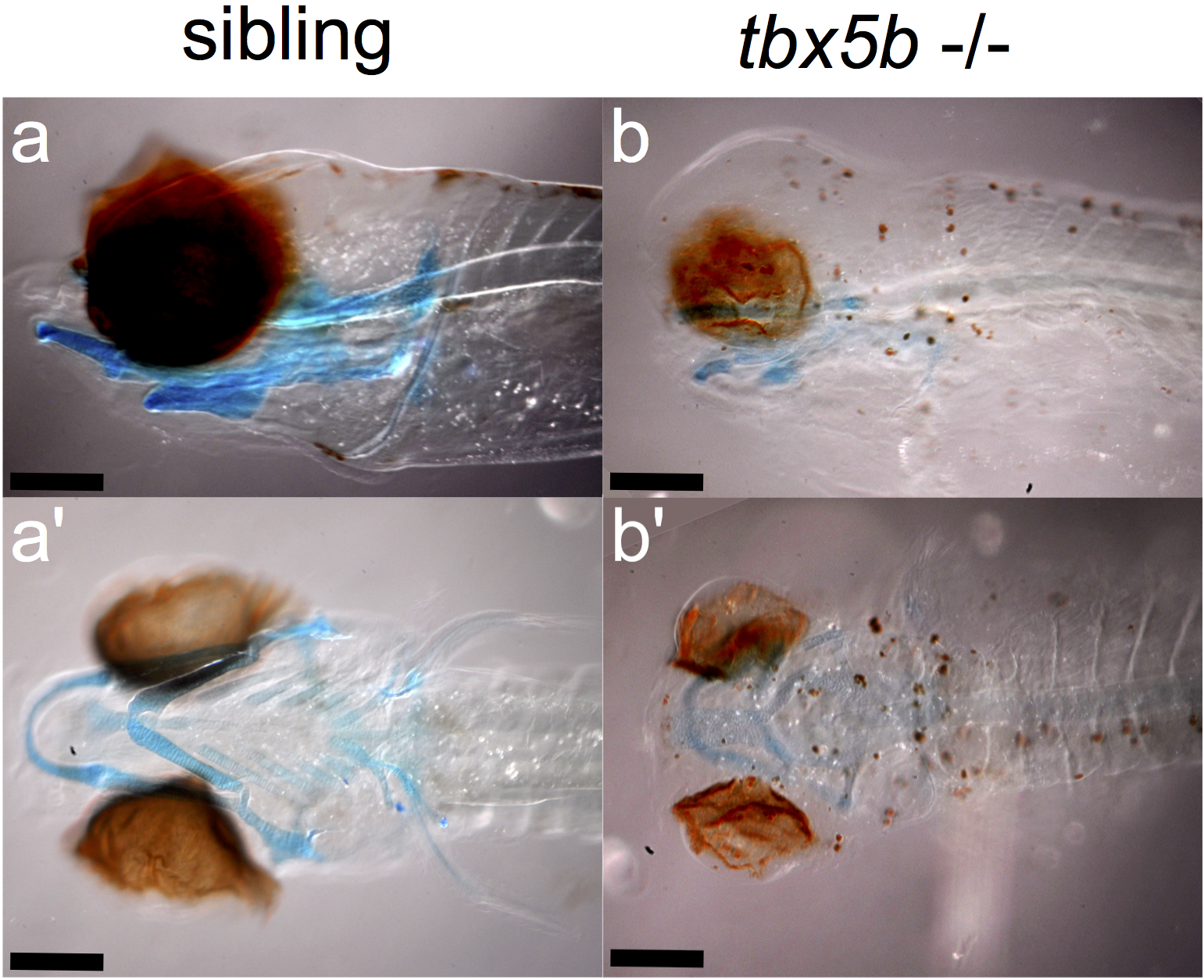

Supplement: S2 Fig — Wildtype siblings show a more protruding lower law that extends beyond the eye as can be seen both from a lateral (a) and ventral (a’) view. Affected tbx5b -/- mutant embryos have a rounder head and a jaw that does not protrude beyond the eyes, as seen from both a lateral (b) and ventral (b’) view. Scalebar is 100 μm. (TIFF) [file pone.0208766.s002.tiff]

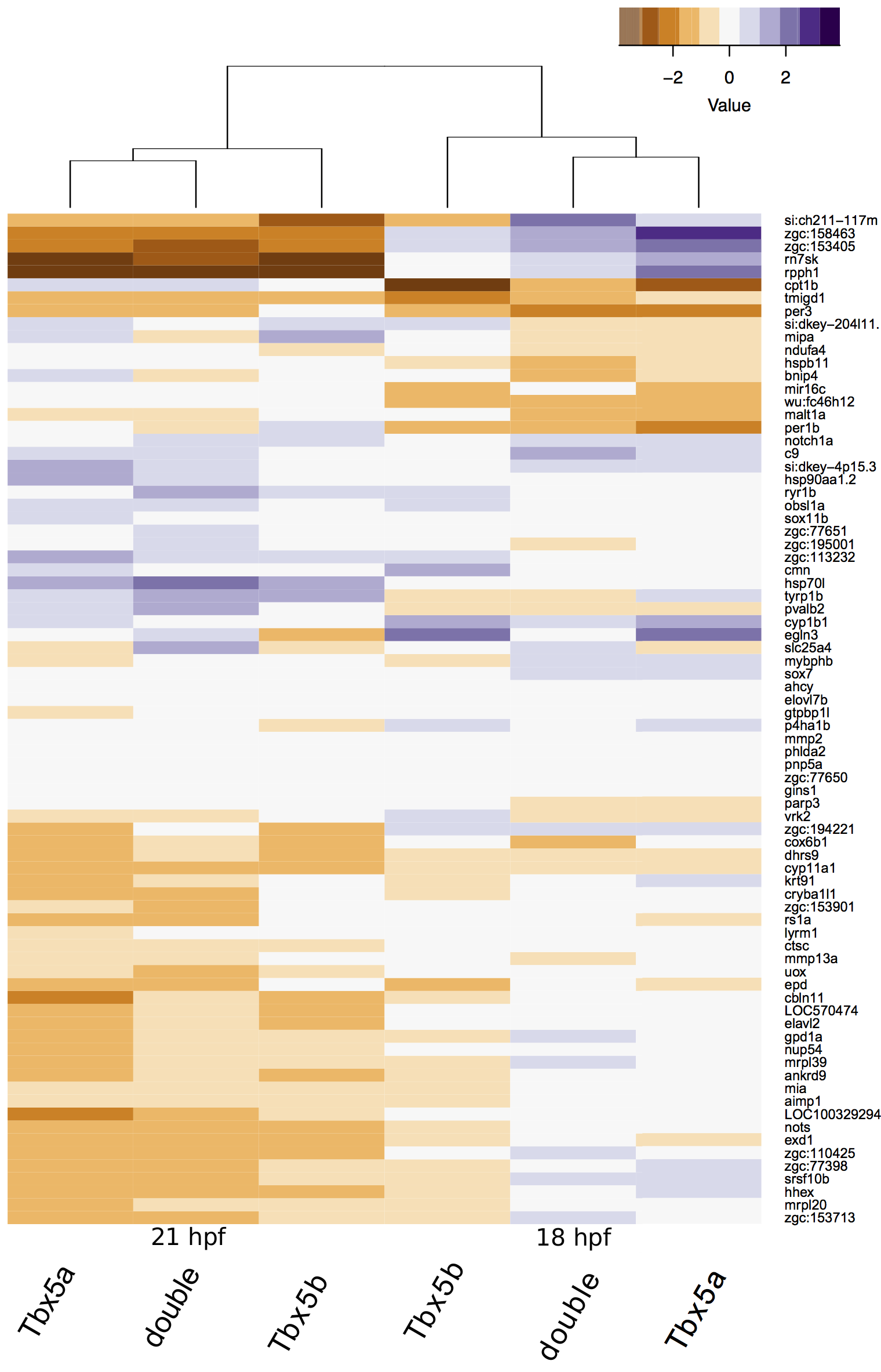

Supplement: S3 Fig — Expression changes compared to wildtype embryos at each time point for all genes that were identified using 2 or more differential gene expression methods. Differential gene expression values were determined using the Cuffdiff data for this figure. (TIFF) [file pone.0208766.s003.tiff]

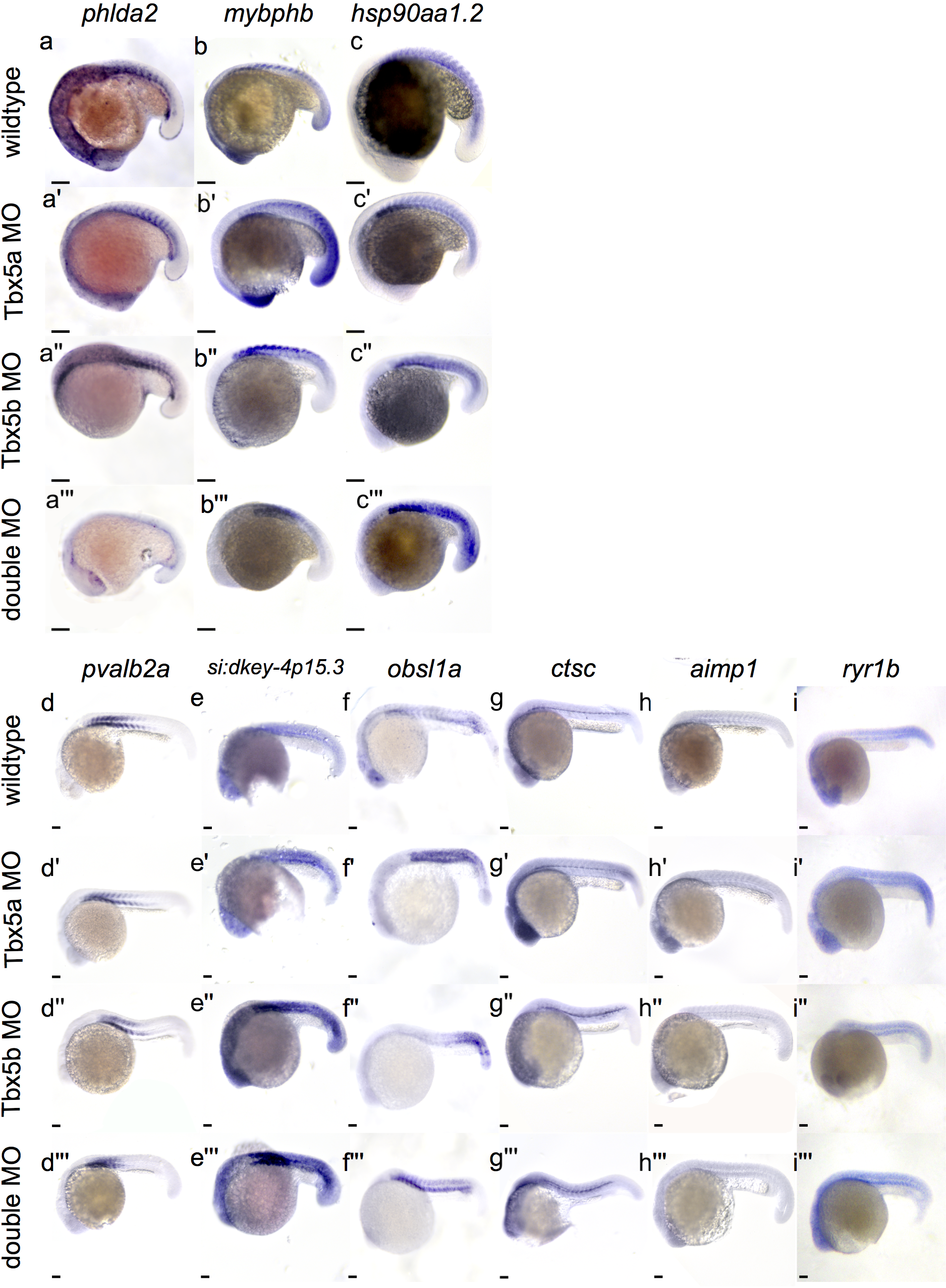

Supplement: S4 Fig — In situ hybridization of all genes differentially expressed in the somites. (a-a”’) At 18hpf, phlda2 is upregulated in Tbx5a-deficient (a’) and Tbx5b-deficient (a”) embryos compared to wildtype embryos (a’), but downregulated in the double-deficient embryos (a”’). At 18hpf, mybphb shows upregulation in Tbx5a-deficent (b’) and double-deficient (b”’) embryos compared to wildtype embryos (b). At 18hpf, hsp90aa1.2 is upregulated in Tbx5a-deficient (c’), Tbx5b-deficient (c”) and double-deficient (c”’) embryos compared to wildtype (c) embryos, especially in the anterior somites. At 21 hpf, pvalb2 expression is increased in Tbx5b-deficient (d”) and double-deficient (d”’) embryos compared to wildtype (d) but not Tbx5a-deficient embryos (d’). At 21 hpf, si:dkey-4p:15.3 expression is increased in Tbx5a-deficient (e’), Tbx5b-deficient (e”) and double-deficient (e”’) embryos. At 21 hpf, obsl1a expression is upregulated in Tbx5a-deficient (f’), Tbx5b-deficient (f”) and double-deficient (f”’) embryos compared to wildtype (f) embryos. At 21 hpf, ctsc expression is expanded in Tbx5a-deficient (g’) and Tbx5b-deficient (g”) embryos compared to wildtype (g) embryos. At 21 hpf, aimp1 expression is decreased in Tbx5a-deficient (h’), Tbx5b-deficient (h”) and double-deficient (h”’) embryos compared to wildtype embryos (h). At 21 hpf, ryr1b expression is increased in both Tbx5a-deficient (i’) and double-deficient (i”’) embryos compared to wildtype (i) embryos. (j-k) Comparison of length between Tbx5b mutant embryos and siblings, n = 10, measurements in μm. (j) Tbx5b-deficient embryos at 3 dpf are significantly shorter than their siblings. (k) Somite size is not significantly different at 3dpf between Tbx5b-deficient embryos and their wildtype siblings. Since somite size varies along the AP axis, measurements were taken of the more anterior somites only. (l) At 25 hpf, there is a significant difference in somite number between wildtype and Tbx5b morpholino injected embryos. Scale bar is 100 [file pone.0208766.s004.tiff]

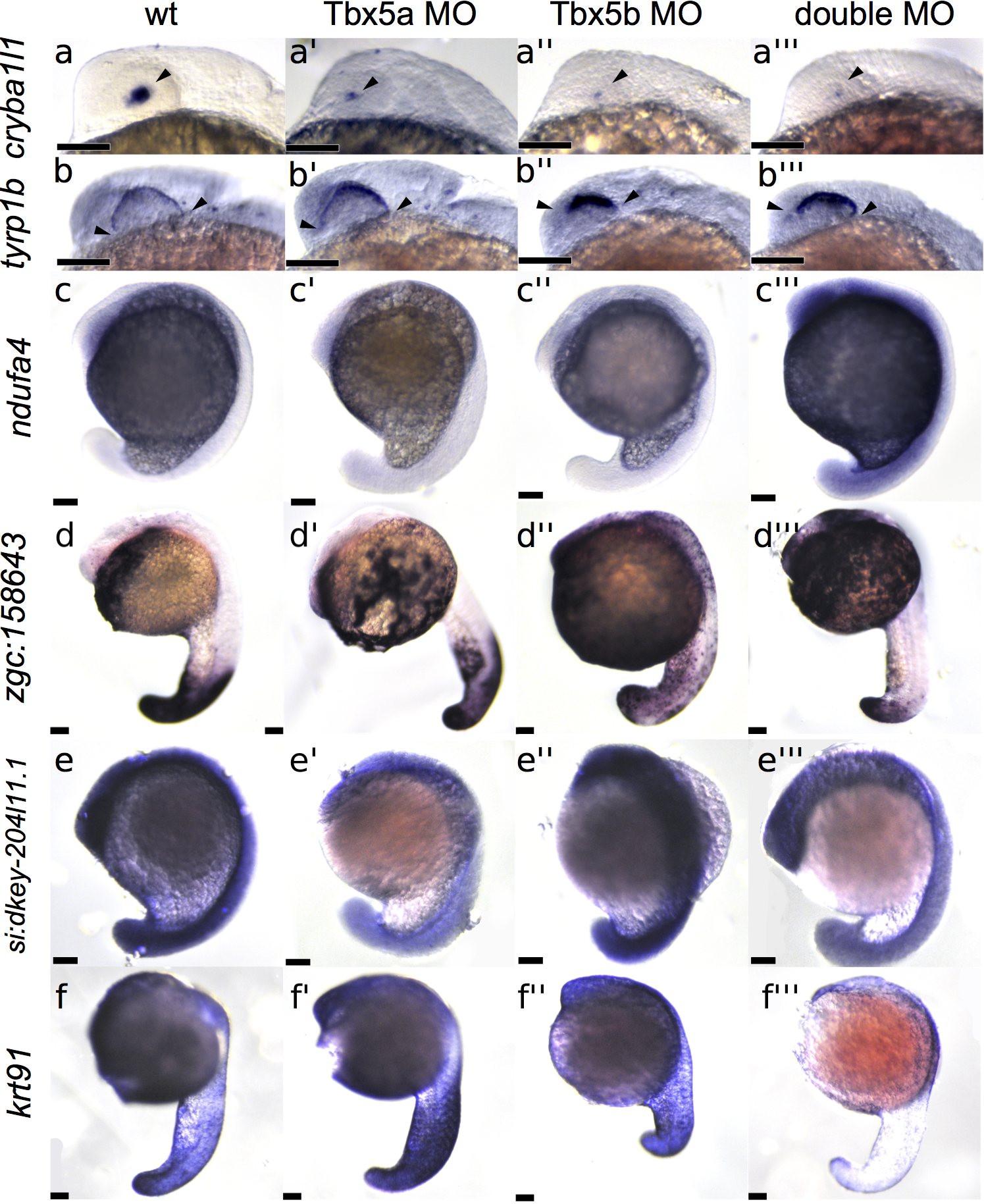

Supplement: S5 Fig — All views are lateral. At 21 hpf, cryba1l1 is downregulated in Tbx5a-deficient (a’), Tbx5b-deficient (a”), and double-deficient (a”’) embryos compared to wildtype (a) embryos in the eye. Arrowheads mark the expression. At 21 hpf, tyrp1b expression is expressed at higher levels in Tbx5a-deficient(b’), Tbx5b-deficient (b”) and double-deficient (b”’) eyes compared to wildtype (b) eyes. Arrowheads mark the limits of the expression domain. At 18 hpf, ndufa4 is upregulated in the double-deficient embryo (c”’) compared to wildtype (c) embryos. Expression of zgc158642 at 21 hpf is upregulated in Tbx5b-deficient (d”) and double-deficient (d”’) embryos compared to wildtype embryos (d). At 18 hpf, si-dkey-204l11.1 expression is downregulated in Tbx5a-deficient (e’) and double-deficient (e”’) embryos compared to wildtype (e) embryos. At 21 hpf, expression of krt91 is downregulated in double-deficient embryos (f”’) compared to wildtype embryos (f). Note f” has normal tail length, but it is bent out of focus of this image. Scale bars are 100 μm. (TIFF) [file pone.0208766.s005.tiff]
